# Development and external validation of a machine learning-based model for identifying advanced Parkinson’s disease

**DOI:** 10.3389/fnagi.2026.1867251

**Published:** 2026-06-03

**Authors:** Xiao-Ru Tan, Shao-Dan Zhou, Bing-Hua Lv, Fu-Zan Wei, Wen-Bin Teng, Chao Qin, Rui-Ting Hu

**Affiliations:** 1 Department of Neurology, The First Affiliated Hospital of Guangxi Medical University, Nanning, China; 2 Department of Neurology, Minzu Hospital of Guangxi Zhuang Autonomous Region, Nanning, China

**Keywords:** advanced stage, biomarkers, machine learning, Parkinson’s disease, predictive model

## Abstract

**Background:**

Identifying patients with advanced stage in Parkinson’s disease (PD) is crucial for timely therapeutic intervention, yet current tools rely on subjective clinical scales or expensive biomarkers. We aimed to develop and validate a predictive model based on routine blood biomarkers selected by machine learning algorithms.

**Methods:**

We retrospectively analyzed data from 536 PD patients in a discovery cohort and 80 patients in an independent external validation cohort. Patients were classified as having early or advanced stage based on Hoehn and Yahr staging scale. LASSO and Random Forest (RF) algorithms were employed to screen predictors from demographic and routine blood variables. Selected features were integrated into a multivariable logistic regression model to construct a predictive model. Model performance was evaluated using the AUC, calibration curves, and decision curve analysis (DCA).

**Results:**

Six biomarkers including total bilirubin (TB), indirect bilirubin (IBIL), albumin (ALB), cholinesterase (ChE), lactate dehydrogenase (LDH), and creatine kinase (CK), were identified as independent predictors based on LASSO and RF algorithms. The predictive model demonstrated excellent discriminative ability in the discovery cohort (AUC = 0.873) and maintained robust performance in the external validation cohort (AUC = 0.736). Calibration curve showed good agreement between predicted probabilities and observed outcomes. DCA confirmed the clinical net benefit of the model across a wide range of threshold probabilities. Notably, advanced-stage patients exhibited significantly higher levels of TB, IBIL, ALB, and ChE, but lower levels of LDH compared to early-stage patients.

**Conclusion:**

We established a reliable, non-invasive, and economically efficient predictive model using six blood biomarkers to identify advanced PD.

## Introduction

Parkinson’s disease (PD) is the second most common neurodegenerative disorder worldwide (Zhu et al., 2024), characterized by a progressive loss of dopaminergic neurons and the accumulation of α-synuclein (Yusuf et al., 2025). While early-stage PD is often manageable with pharmacological therapy, the progression to advanced stages marks a critical turning point associated with severe motor complications, debilitating non-motor symptoms, and a dramatic decline in quality of life (Huang et al., 2025). The transition to advanced disease significantly increases the burden on caregivers and healthcare systems, often necessitating complex interventions such as deep brain stimulation (DBS) or palliative care (Nuzzo et al., 2025). Consequently, the ability to identify patients at high risk of advanced stage is of paramount importance for optimizing therapeutic strategies, timing surgical interventions, and implementing personalized management plans (Kleinholdermann et al., 2025).

Currently, the assessment of PD stage relies heavily on clinical rating scales, such as the Movement Disorder Society-Unified Parkinson’s Disease Rating Scale (MDS-UPDRS) and the Hoehn and Yahr (H&Y) staging system (Goetz et al., 2004). However, these tools are subjective, susceptible to rater variability, and often reflect disease status only after significant neuronal loss has already occurred. Although promising biomarkers have been identified in cerebrospinal fluid and through advanced neuroimaging, their clinical utility is limited by high costs, invasiveness, and limited availability in primary care settings (Ginanneschi et al., 2025; Shigemoto et al., 2025). There is, therefore, an urgent unmet need for accessible, non-invasive, and cost-effective biomarkers that can identify disease trajectory in routine clinical practice.

Routine blood biochemical tests including markers of liver function, lipid metabolism, and enzymatic activity, represent an underutilized resource in PD prognostication. These tests are inexpensive, widely available, and routinely performed during standard patient workups. Emerging evidence suggests that systemic metabolic alterations, such as oxidative stress (Alqahtani et al., 2023), mitochondrial dysfunction (Henrich et al., 2023), and chronic inflammation (Patani et al., 2023), are intimately linked to PD pathogenesis and may be reflected in peripheral blood profiles. For instance, alterations in bilirubin (Jayanti et al., 2021), albumin (Jia et al., 2024), and various enzymes (Laugisch et al., 2024; Gao et al., 2025) have been individually associated with PD severity in cross-sectional studies. However, findings remain inconsistent, and most studies have focused on single biomarkers or used traditional statistical methods that fail to capture complex, non-linear interactions between multiple variables. Furthermore, few studies have developed and externally validated a comprehensive predictive model integrating these routine markers.

Recent advances in machine learning algorithms offer a powerful approach to overcome these limitations. Unlike conventional regression models, machine learning algorithms can effectively handle high-dimensional data, reduce overfitting, and identify robust feature subsets from noisy clinical datasets (Akter and Abdullah, 2026). By combining the feature selection strength with the interpretability of traditional logistic regression, it is possible to construct a highly accurate yet clinically practical prediction tool. In this study, we aimed to develop and validate a novel predictive model based on routine blood biomarkers to identify advanced PD. We employed a dual-machine learning strategy to screen for the most robust predictors from a wide range of clinical variables in the large discovery cohort. Then the performance and generalizability of the resulting model were rigorously evaluated in an independent external validation cohort. Our goal is to develop and validate a novel model based on routine blood biomarkers to identify patients with advanced PD.

## Materials and methods

### Study population and design

This study recruited a total of 616 patients diagnosed with PD across two medical centers between February 2022 and December 2024. The discovery cohort comprised 536 individuals (316 males and 220 females) admitted to or visiting the outpatient clinics of the First Affiliated Hospital of Guangxi Medical University. An independent external validation cohort consisting of 80 PD patients was assembled from the Minzu Affiliated Hospital of Guangxi Medical University. All participants fulfilled the diagnostic criteria established by the 2015 Movement Disorder Society (MDS) for PD (Postuma et al., 2015). Disease severity was assessed using the Hoehn and Yahr (H&Y) staging scale (Goetz et al., 2004), stratifying patients into an early stage (H&Y stages 0–2.5) and an advanced stage (H&Y stages ≥ 3). The study protocol received approval from the Institutional Ethics Committee and adhered strictly to the principles of the Declaration of Helsinki.

### Eligibility criteria

Participants were included if they met the MDS Clinical Diagnostic Criteria for PD and possessed complete H&Y staging records. Exclusion criteria encompassed: (1) severe comorbidities involving the heart, liver, or kidneys; (2) presence of dementia, malignancies, gastrointestinal pathologies, autoimmune disorders, or thyroid dysfunction (hyper- or hypothyroidism); and (3) other significant chronic conditions. Additionally, individuals with incomplete clinical documentation or missing laboratory data were excluded from the final analysis.

### Clinical data collection

Demographic and clinical variables were extracted from medical records including age, sex, and history of comorbidities (e.g., diabetes mellitus, chronic kidney disease, cardiovascular disease). A comprehensive panel of routine blood biomarkers was collected, covering: Hematological parameters: White blood cell count, hemoglobin, platelet count, and lymphocyte count. Hepatic function: Albumin (ALB), total protein, alanine aminotransferase (ALT), aspartate aminotransferase (AST), total bilirubin (TB), and indirect bilirubin (IBIL). Renal function and others: Creatinine, procalcitonin, and prealbumin (PA). Data integrity was verified by two independent researchers. Missing data were present in several baseline variables across all cohorts. The overall missingness rate ranged from 0 to 12%. To quantify this, we first calculated the proportion of missing values for each variable in the discovery and external validation cohorts separately. No variable exceeded 20% missingness, and no participant had more than 15% of their data missing. Multiple imputation by chained equations (MICE) was performed in R software. Imputation models included all predictor variables used in the final model to ensure compatibility with the analysis model. Predictive mean matching (PMM) was used for continuous variables, and logistic regression for binary variables.

### Feature selection via machine learning

Prior to feature selection, continuous variables were standardized using Z-score transformation. To isolate the most robust predictors of PD stage, we implemented a dual-machine learning approach: Least Absolute Shrinkage and Selection Operator (LASSO) Regression: Utilizing a binomial logistic loss function, LASSO regression was performed with 10-fold cross-validation to identify the optimal regularization parameter (λ) that minimized deviance. Random Forest (RF): An RF model comprising 500 decision trees was constructed. Variable importance was ranked based on the Gini index, and the top 10 features exhibiting the greatest mean decrease in accuracy were retained. Variables consistently identified by both LASSO and RF algorithms were deemed robust candidates. These overlapping features were subsequently entered into a multivariable logistic regression model to determine independent predictors (significance level set at *P* < 0.05).

### Predictive model construction and evaluation

A predictive nomogram was constructed based on the independent biomarkers identified through the machine learning pipeline. The discovery cohort was used for model development and internal validation. We performed 10-fold cross-validation to evaluate the model’s performance and mitigate the risk of overfitting. The final internal performance metrics reported are the mean values obtained from the 10 cross-validation folds. Model predictive performance was determined by receiver operating characteristic (ROC) curves, with the area under the curve (AUC) calculated for each cohort. We also employed calibration curve to compare observed and predicted probabilities. The Brier score (where lower values indicate superior accuracy), and the Hosmer-Lemeshow goodness-of-fit test (*P* > 0.05 suggests adequate fit) were applied to determine the accuracy and robustness of the model. Determined through Decision Curve Analysis (DCA) to quantify the net benefit across various threshold probabilities.

### Nomogram development and mathematical formulation

A predictive nomogram was constructed based on the six independent predictors from the final multivariable logistic regression model. The points assigned to each predictor in the nomogram are proportional to its beta coefficient (β). The total points for an individual are calculated as the sum of the points for each of their six biomarker values. The predicted probability of being in the advanced PD stage is then derived from the total points using the logistic function: P (Advanced PD) = 1/(1 + exp (-(β_0_ + β_1_*TB + β_2_*IBIL + β_3_*ALB + β_4_*ChE + β_5_*LDH + β_6_*CK))). Where β_0_ is the model intercept and β_1_ to β_6_ are the coefficients for the respective biomarkers. For example, for a patient with TB = 12 μmol/L, IBIL = 4 μmol/L, ALB = 38 g/L, ChE = 7500 U/L, LDH = 160 U/L, and CK = 80 U/L, the total points would be 273, corresponding to a predicted probability of 0.793.

### Statistical analysis

All statistical computations were executed using R software (version 4.3.0). Continuous variables following a normal distribution were expressed as mean ± standard deviation (SD), whereas non-normally distributed data were presented as median with interquartile range (IQR). Categorical variables were summarized as frequencies and percentages. Group comparisons for normally distributed continuous variables with homogeneous variances were conducted using independent samples *t*-tests; Welch’s *t*-test was applied when variance homogeneity was violated. Non-parametric tests were utilized for data deviating from normality. Differences in categorical variables were assessed using Chi-square tests or Fisher’s exact tests, as appropriate. A two-sided *P*-value of less than 0.05 was considered statistically significant.

## Results

### Participant characteristics

**FIGURE 1:**
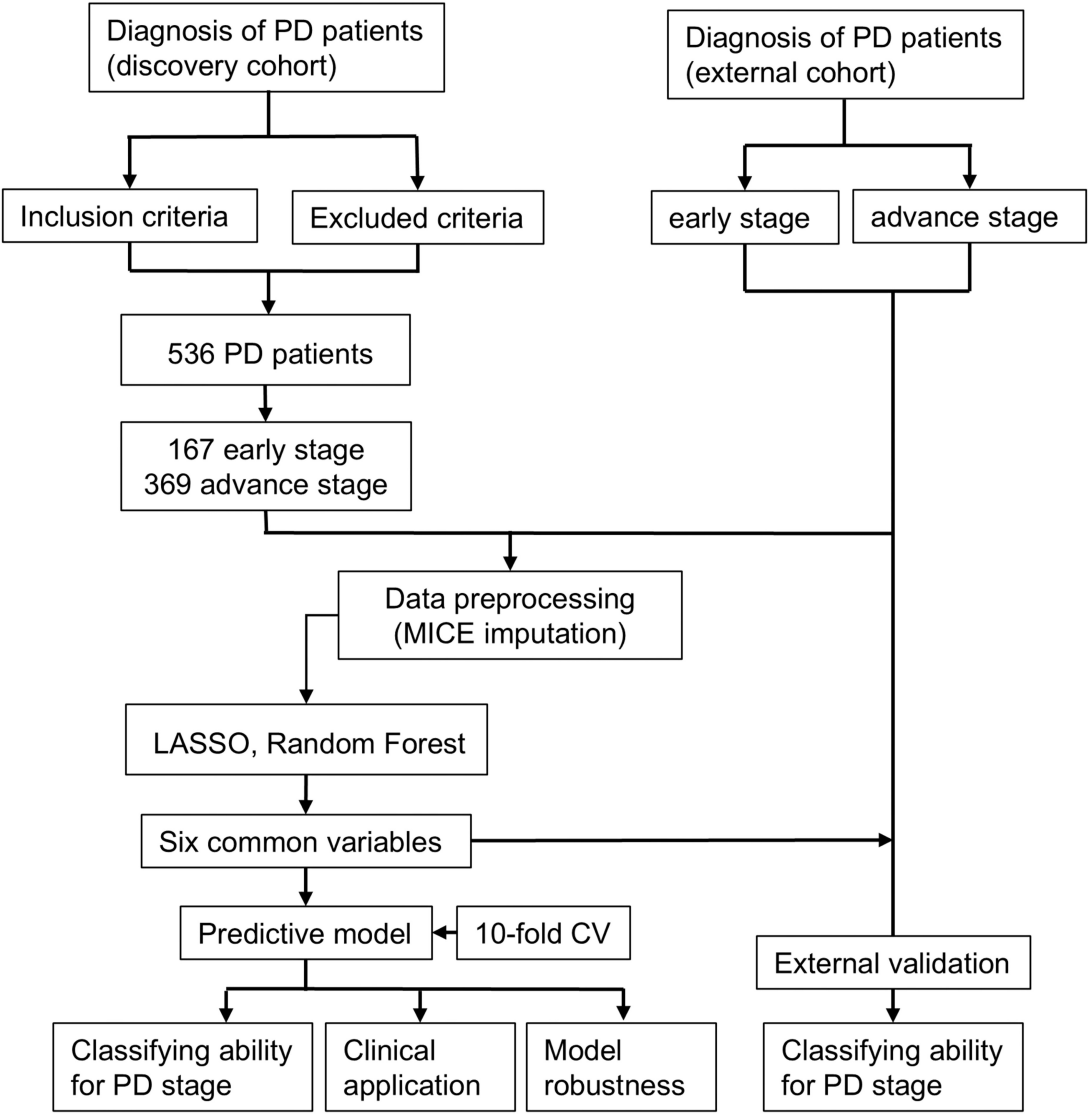
Workflow of the study.

The study design, data collection, and analysis workflow are illustrated in Figure 1. The study analyzed consecutive 536 PD patients from the center as the discovery cohort. This cohort had a mean age of 67.5 ± 10.8 years and included 316 (59.0%) males patients. Based on the Hoehn-Yahr stage, patients were categorized into early stage (H&Y score < 3) and advanced stage (H&Y score ≥ 3), which included 167 and 369 PD patients, respectively.

Comparison of demographic and clinical variables between early and advanced stages revealed significant differences. Patients in the advanced stage were significantly older than those in the early stage (median age: 71.0 vs. 68.0 years, *P* < 0.001). Regarding laboratory biomarkers, the advanced stage group exhibited significantly higher levels of total bilirubin (TB), indirect bilirubin (IBIL), direct bilirubin (DBIL), albumin (ALB), cholinesterase (ChE), prealbumin (PA), neutrophil count (NEU), total protein (TP), and A/G ratio (all *P* < 0.05). Conversely, levels of red blood cell (RBC), hemoglobin (HB), lymphocyte count, globulin (GLB), lactate dehydrogenase (LDH), α-hydroxybutyrate dehydrogenase (α-HBD), and alkaline phosphatase (ALP) were significantly lower in the advanced stage group (all *P* < 0.05). No significant differences were observed in sex distribution, smoking status, drinking status, or comorbidities including diabetes and hypertension (all *P* > 0.05). Additionally, white blood cell (WBC) count, creatine kinase (CK), and liver enzymes (AST, ALT) did not differ significantly between the two groups. The detailed clinical characteristics are presented in Table 1.

**TABLE 1:** Characteristics of the included PD patients.

Variables	All	Early stage (*n* = 167)	Advanced stage (*n* = 369)	*P*-value
Age (years)	68.0 (60.0–75.0)	68.0 (60.0–73.0)	71.0 (63.5–77.5)	< 0.001
Sex				
Male	316 (59.0%)	99 (59.3%)	217 (58.8%)	0.993
Female	220 (41.0%)	68 (40.7%)	152 (41.2%)	
Smoking	122 (22.8%)	36 (21.6%)	86 (23.3%)	0.737
Drinking	109 (20.3%)	29 (17.4%)	80 (21.7%)	0.301
Heart failure	7 (1.3%)	4 (2.4%)	3 (0.8%)	0.279
Atrial fibrillation	48 (9.0%)	21 (12.6%)	27 (7.3%)	0.070
Hypertension	270 (56.2%)	94 (62.7%)	176 (53.3%)	0.070
Diabetes	107 (22.3%)	39 (23.3%)	68 (18.4%)	0.238
WBC (× 10^9^/L)	6.6 (5.4–8.2)	6.5 (5.3–8.1)	6.7 (5.6–8.5)	0.125
RBC (× 10^12^/L)	4.2 (3.8–4.6)	4.3 (4.0–4.7)	4.0 (3.5–4.3)	< 0.001
HB (g/L)	123.0 (111.0–133.0)	125.7 (113.8–135.4)	115.7 (104.2–126.0)	< 0.001
Platelet (× 10^9^/L)	232.9 (189.0–279.0)	228.1 (189.0–275.0)	243.0 (190.5–285.8)	0.116
NEU (× 10^9^/L)	4.2 (3.2–5.6)	4.0 (3.1–5.3)	4.4 (3.5–6.4)	0.003
Lymphocytes (× 10^9^/L)	1.5 (1.2–1.9)	1.6 (1.2–2.0)	1.4 (1.1–1.7)	< 0.001
TB (μmoL/L)	10.6 (8.4–13.3)	8.2 (6.2–10.1)	12.0 (9.5–15.1)	< 0.001
IBIL (μmoL/L)	3.2 (2.5–4.1)	2.7 (2.2–3.5)	3.5 (2.8–4.3)	< 0.001
DBIL (μmoL/L)	7.5 (5.5–9.6)	5.5 (3.7–7.1)	8.3 (6.5–11.2)	< 0.001
I/D ratio	0.3 (0.2–0.4)	0.3 (0.3–0.4)	0.3 (0.2–0.3)	< 0.001
TP (g/L)	65.8 (61.8–69.7)	65.1 (61.2–68.3)	66.1 (62.0–70.5)	0.043
ALB (g/L)	37.3 (34.8–39.6)	35.8 (32.3–38.1)	38.0 (35.6–39.9)	< 0.001
GLB (g/L)	28.4 (25.4–31.6)	29.0 (26.1–32.3)	27.9 (24.8–31.3)	0.033
A/G ratio	1.3 (1.2–1.5)	1.2 (1.1–1.4)	1.4 (1.2–1.5)	< 0.001
AST (U/L)	22.0 (17.0–27.0)	22.0 (18.0–28.0)	22.0 (17.0–27.0)	0.852
ALT (U/L)	14.5 (10.5–21.0)	15.0 (9.0–22.0)	14.0 (11.0–20.0)	0.772
AST/ALT ratio	1.4 (1.1–2.0)	1.5 (1.1–2.2)	1.4 (1.1–1.9)	0.192
ALP (U/L)	72.0 (60.0–88.0)	79.0 (64.0–97.0)	70.0 (59.0–84.0)	< 0.001
PA (mg/L)	228.8 (189.9–271.4)	210.4 (162.0–255.7)	235.7 (200.8–277.8)	< 0.001
ChE (U/L)	7072.5 (5858.5–8415.0)	6602.4 (4949.0–7677.5)	7402.0 (6272.0–8606.0)	< 0.001
CK (U/L)	78.0 (52.0–112.5)	72.0 (43.5–128.6)	78.0 (55.0–107.0)	0.201
CK-MB (U/L)	14.0 (11.0–17.5)	14.0 (11.0–19.0)	14.0 (12.0–17.0)	0.586
LDH (U/L)	172.0 (147.0–203.0)	187.0 (153.0–214.5)	167.0 (146.0–187.0)	< 0.001
α-HBD (U/L)	122.5 (106.0–147.2)	137.0 (107.0–158.5)	121.0 (105.0–138.0)	< 0.001

WBC, white blood cell; NEU, neutrophil; RBC, red blood cell; HB, hemoglobin; CK, creatine kinase; CK-MB, creatine kinase isoenzymes; LDH, lactate dehydrogenase; TBIL, total bilirubin; DBIL, Direct bilirubin; IBIL, Indirect bilirubin; I/D ratio, IBIL/DBIL ratio; TP, total protein; ALB, Albumin; GLB, globulin; A/G ratio, ALB/GLB ratio; PA, prealbumin; ChE, cholinesterase.

For external validation, we enrolled 80 PD patients from another center at the same period and divided the patients based on the stage of the PD (40 early stage and 40 advanced stage). The mean age of patients, and the number of gender were similar to the discovery cohort. Detailed demographic and clinical comparisons are shown in Supplementary Table S1.

### Screen of variables key to the PD stage by machine learning methods

**FIGURE 2:**
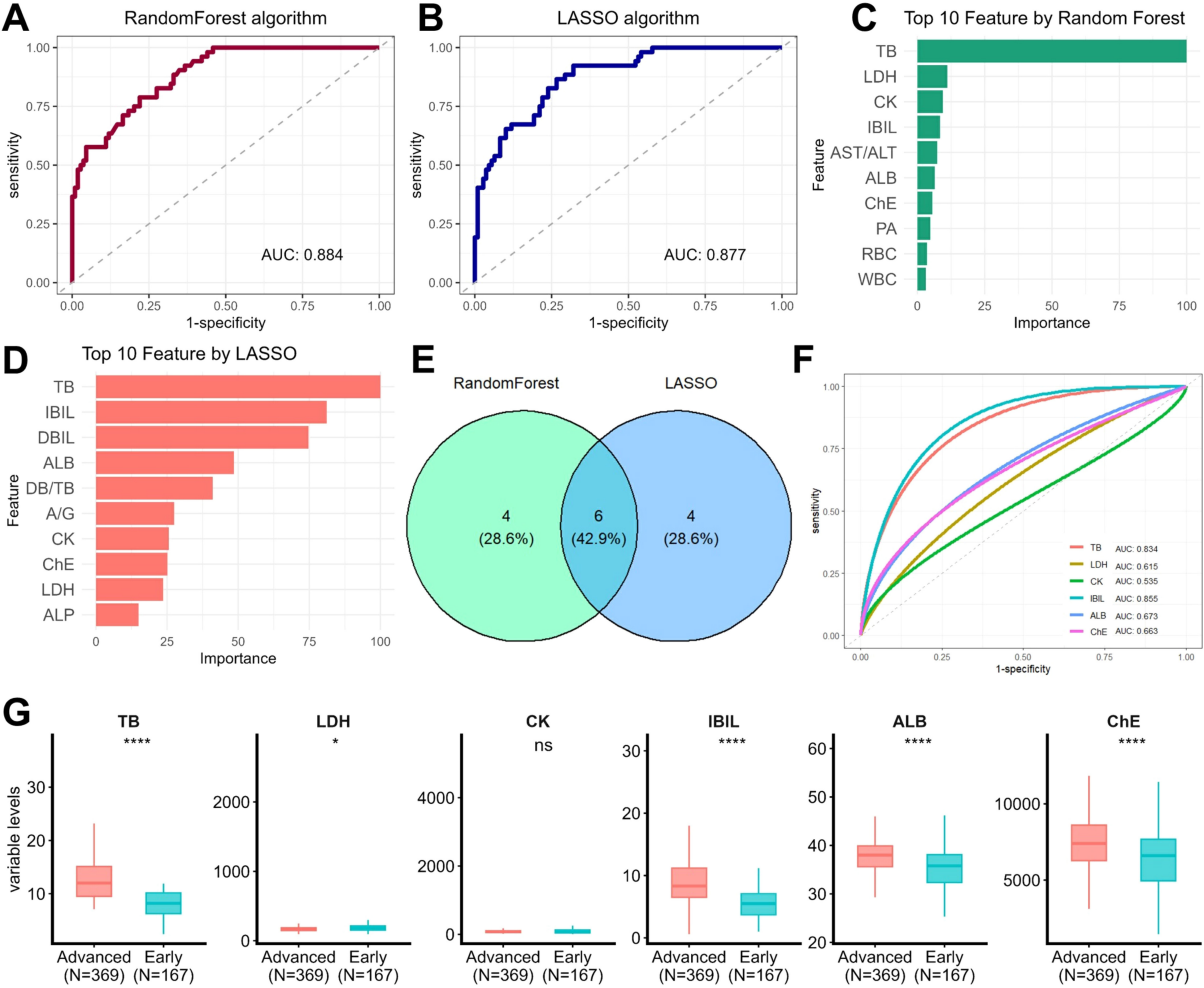
Screen of variables key to the PD stage by machine learning methods. **(A)** Predictive value of PD stage by Random Forest model. **(B)** Predictive value of PD stage by LASSO model. **(C)** Top 10 variables screened by Random Forest model. **(D)** Top 10 variables screened by LASSO model. **(E)** Venn plot for the common variables of Random Forest model and LASSO model. **(F)** Predictive value of six variables for PD stage. **(G)** Comparison of six variables levels between early- and advanced- stage of PD. *p < 0.05, ****p < 0.0001, ns, not significant.

To screen the variables related to the PD stage, we used two machine learning methods including Random Forest and LASSO models, we found that these two methods showed high predictive value on the stage of PD, with AUC value as 0.884 and 0.877 in Random Forest and LASSO models (Figures 2A,B). These two models screen several key variables to the PD stage, and the top 10 variables of the two models (Figures 2C,D). Then we overlapped these variables and found 6 common variables including TB, LDH, CK, IBIL, ALB, ChE (Figure 2E). To be noted, CK was identified as a key predictor by machine learning despite no significant univariate difference. The predictive value of each variable was showed in Figure 2F. The levels of the six variables between early and advanced stage of PD were showed in Figure 2G. These results suggested these six variables are crucial to the PD stage.

### Establishment of predictive model for the PD stage

**FIGURE 3:**
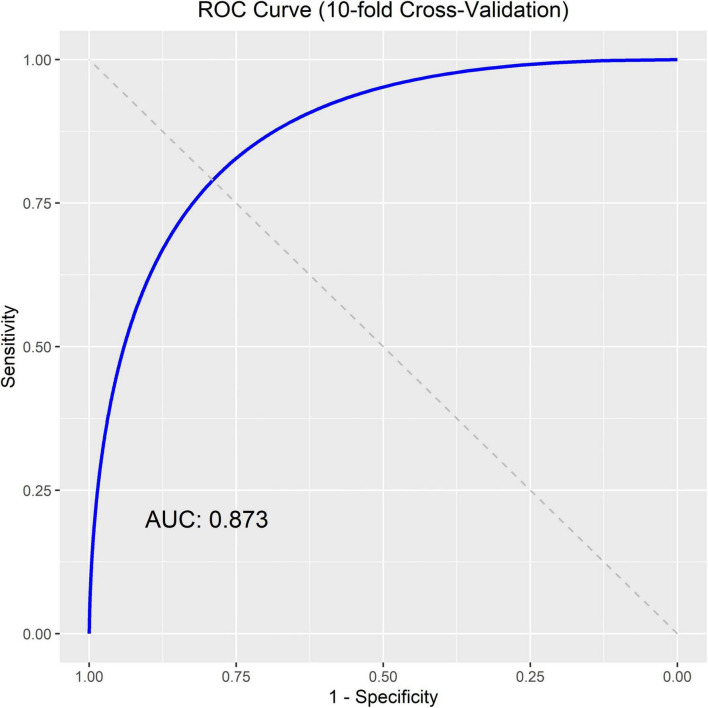
Establishment of predictive model for the PD stage. Predictive value of the model for PD stage in the discovery cohort by 10-fold cross-validation in multivariate logistic regression analysis.

We establish a predictive model for the PD stage based on the six selected biomarkers (TB, IBIL, ALB, ChE, LDH, and CK) by multivariate logistic regression analysis. Using 10-fold cross-validation, the model achieved a mean AUC of 0.873 (95% CI: 0.834–0.896) (Figure 3). The sensitivity, specificity, PPV, and NPV were 0.868 (95% CI: 0.814–0.916), 0.667 (95% CI: 0.618–0.713), 0.919 (95% CI: 0.887–0.947), and 0.541 (95% CI: 0.502–0.584), respectively. These results indicated that the six-variable model has good classifying performance for the PD stage.

### Nomogram construction and evaluation

**FIGURE 4:**
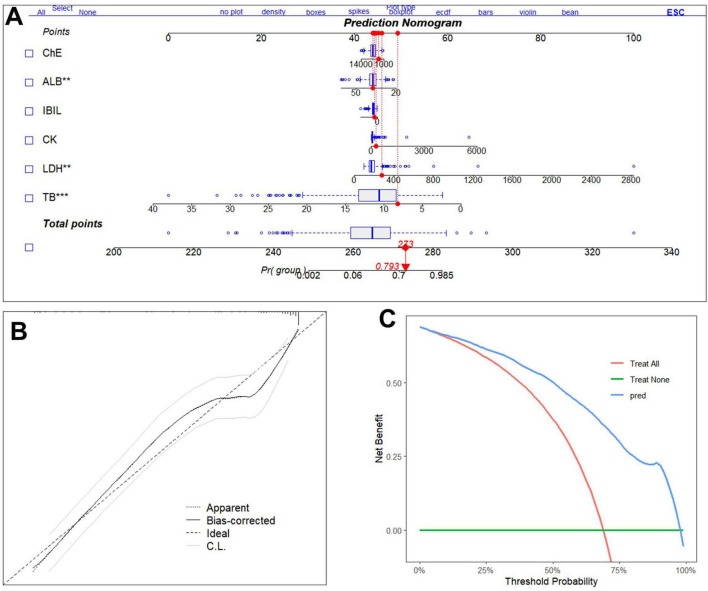
Nomogram construction and evaluation. **(A)** Nomogram for the predictive model and clinical parameters. Each independent predictor of Nomogram was mapped to a “points” value at the top of the nomogram. **(B)** Calibration curve for the nomogram was used to evaluate the consistency between model predicted probabilities and actual observed probabilities. **(C)** Decision curve Analysis (DCA) assess the net benefit of the nomogram across different high-risk thresholds.

The logistic regression model was built using Z-score standardized versions of the six biomarkers. Therefore, the beta coefficients correspond to a one standard deviation change in the standardized variables. The six predictors were initially identified by LASSO and RF, and then confirmed as independent predictors by multivariate logistic regression (Table 2). Based on the six independent predictors identified by the multivariate logistic regression model, we constructed a nomogram to predict the probability of advanced PD stage (Figure 4A). Each predictor is assigned a score on the points scale based on its regression coefficient. The sum of these scores (Total Points, which represents the individual’s risk score) corresponds to the predicted probability of disease stage on the bottom axis. The nomogram demonstrated excellent discrimination, with a bootstrap-corrected C-index of 0.880. Calibration analysis revealed good agreement between predicted and observed advanced stage probabilities for discover cohort (Figure 4B), supported by a low Brier score of 0.027 and the Hosmer-Lemeshow test confirmed adequate model calibration (*P* = 0.154). DCA curve showed a positive net benefit across a wide range of threshold probabilities (Figure 4C), indicating robust clinical utility.

**TABLE 2:** Multivariate logistic regression for the six predictors.

Variables	β value	OR (95%CI)	*P*-value
TB	0.514	1.672 (1.531–1.890)	< 0.001
LDH	−0.006	0.994 (0.990–0.998)	< 0.001
CK	−0.001	0.999 (0.997–1.001)	0.183
IBIL	0.0363	1.037 (1.015–1.059)	< 0.001
ALB	0.089	1.093 (1.065–1.122)	< 0.001
ChE	0.001	1.001 (1.000–1.002)	< 0.001

### Validation of the predictive model in an external cohort

**FIGURE 5:**
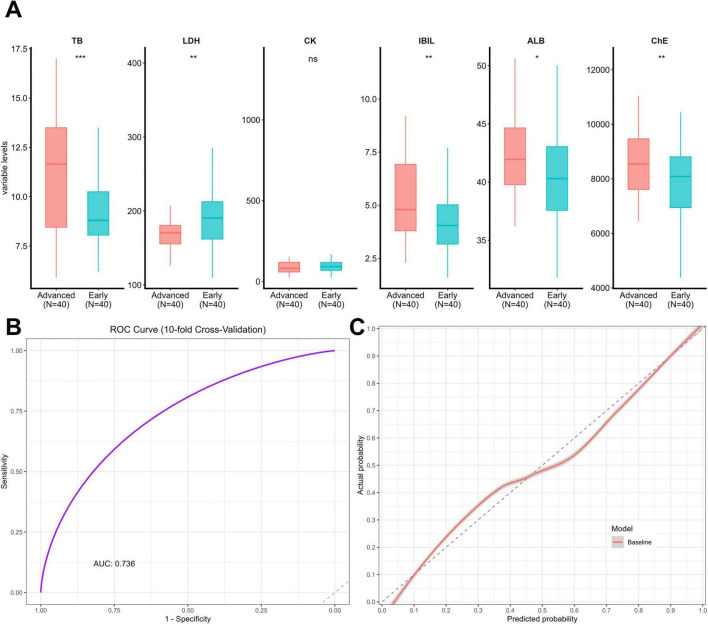
Validation of the predictive model in an external cohort. **(A)** Comparison of six variables levels between early- and advanced- stage of PD in external cohort. **(B)** Predictive value of the six-variable model for PD stage in external cohort by 10-fold cross-validation. **(C)** Calibration curve for the nomogram was used to evaluate the consistency between model predicted probabilities and actual observed probabilities for external cohort.

To validate the clinical utility of the signature, we analyzed serum levels of six variables in an external cohort of 80 patients (40 early stage, 40 advanced stage). The coefficients from the final logistic regression model developed in the discovery cohort were directly applied to the external validation cohort without re-fitting. Consistent with the discovery cohort, the six variables showed significant differences between the two groups in the external cohort, with TB (*p* < 0.001), IBIL (*p* = 0.004), ALB (*p* = 0.032), and ChE (*p* = 0.008) levels being higher, while LDH (*p* = 0.004) levels were lower in advanced patients, but CK levels without statistical significance (*p* = 0.392) (Figure 5A). Combining the six variables using the proposed nomogram approach, the predictive model achieved an AUC of 0.736 (95% CI: 0.632–0.849) (Figure 5B), and the sensitivity, specificity, PPV, and NPV were 0.775 (95% CI: 0.650–0.900), 0.625 (95% CI:0.475–0.775), 0.737 (95% CI:0.619–0.853), and 0.674 (95% CI: 0.583–0.778), demonstrating robust generalizability. The Calibration curve revealed good agreement between predicted and observed advanced stage probabilities (Figure 5C), with a low Brier score of 0.036 and the Hosmer-Lemeshow test confirmed adequate model calibration (*P* = 0.815).

## Discussion

In this study, we successfully developed and validated a novel nomogram integrating six routine blood biomarkers including total bilirubin (TB), indirect bilirubin (IBIL), albumin (ALB), cholinesterase (ChE), lactate dehydrogenase (LDH), and creatine kinase (CK), to predict the advanced stages in PD patients. Our model demonstrated robust discriminative ability with an AUC of 0.873 in the discovery cohort and maintained excellent performance in an independent external validation cohort (AUC = 0.736). Calibration curves and DCA further confirmed the clinical utility of this tool. To our knowledge, this is the first study to combine machine learning algorithms (LASSO and Random Forest) with multivariable logistic regression to identify a metabolic signature for PD stage, offering a cost-effective and accessible strategy for early risk stratification in clinical practice.

Our findings highlight the critical role of oxidative stress and mitochondrial dysfunction in PD stage, as reflected by the selected biomarkers. Bilirubin, traditionally viewed as a waste product, is increasingly recognized as a potent endogenous antioxidant (Ziberna et al., 2016). Consistent with previous reports suggesting altered heme metabolism in PD (Macías-García et al., 2019), we observed significantly elevated levels of TB and IBIL in advanced-stage patients. While some studies have reported lower bilirubin levels in early PD compared to controls (Jin et al., 2020), our results suggest that the upregulation of bilirubin may associate with escalating oxidative stress as the disease advances. Similarly, the significant reduction in LDH levels in advanced patients might related to impaired glycolytic metabolism and mitochondrial complex I dysfunction, which are hallmarks of dopaminergic neuronal death (Smeyne and Smeyne, 2013). The inverse association between LDH and disease stage underscores the energy failure hypothesis in late-stage PD.

Furthermore, nutritional and muscular status emerged as key predictors. Lower levels of ALB in the early stage might reflect systemic inflammatory responses or altered hepatic synthetic function associated with neurodegeneration (Sakakibara et al., 2023). Specifically, ChE is involved in the hydrolysis of acetylcholine; its alteration may mirror the cholinergic deficit that becomes prominent in advanced PD, contributing to cognitive decline and motor fluctuations (van Laar et al., 2011). Interestingly, in our study, we found that CK was identified as a robust predictor by our machine learning models despite lacking significance in univariate analysis. This suggests a non-linear relationship between muscle metabolism and PD stage, potentially linking sarcopenia or subclinical myopathy to disease severity (Gui et al., 2025), a connection that warrants further mechanistic investigation.

The strength of our study lies in the rigorous methodology. Unlike previous studies that relied solely on traditional statistical methods or lacked external validation, we employed two machine learning methods to filter noise and select the most robust features before constructing the logistic regression model. This strategy minimized overfitting and enhanced the generalizability of the nomogram. Crucially, the inclusion of an independent external cohort from a different institution validates the stability of our model across diverse patient populations. The use of routine blood tests makes this nomogram highly practical for resource-limited settings where advanced imaging or cerebrospinal fluid biomarkers are unavailable. Our DCA results demonstrate that the model provides a positive net benefit across this clinically relevant threshold range (approximately 5–40%), and notably throughout the 10–30% range. This means that using the model to guide referrals within this range would lead to more true positive identifications of advanced PD without a disproportionate increase in unnecessary referrals, compared to a strategy of either referring no one or referring everyone. Therefore, the model has the potential to improve clinical decision-making efficiency in this specific scenario.

To our knowledge, this is among the first studies to develop and externally validate an interpretable prediction model for identifying advanced-stage PD using a panel of six routinely measured serum biomarkers. While prior research has explored machine learning approaches, clinical scores, imaging features, or individual blood-based markers for PD diagnosis or stage, our work uniquely combines these six accessible, low-cost laboratory parameters into a validated, clinically deployable tool tailored to the H&Y staging framework. The external validation in an independent cohort further strengthens the generalizability of this specific biomarker combination for this well-defined clinical endpoint.

However, several limitations must be acknowledged. First, this was a retrospective study, which inherently carries the risk of selection bias. Second, while we adjusted for major confounders, unmeasured factors such as dietary intake, medication history (such as the effects of levodopa dosage on biomarkers), and genetic profiles (including GBA or LRRK2 mutations) were not included. Third, the sample size, although adequate for model development, was moderate; larger multicenter prospective studies are needed to further refine the cutoff values. Fourth, while the biological plausibility of the markers is strong, the exact causal mechanisms linking these peripheral blood markers to central neurodegeneration remain to be elucidated through basic science research. Fifth, the H&Y staging scale is inherently subjective and susceptible to inter-rater variability. Consequently, our model does not represent an independent biological definition of disease severity. Our model reflects a surrogate signature correlated with clinician-assigned advanced-stage PD, not a ground-truth biological staging system. Therefore, future studies incorporating more objective measures could help disentangle whether these biomarkers track beyond clinical rating scales. Sixth, the external validation cohort was deliberately balanced, comprising an equal number of early-stage, which does not reflect the true prevalence of advanced PD in routine clinical settings. Consequently, the results from this cohort should be interpreted with caution. Future prospective studies in unselected, population-based cohorts are needed to evaluate the model’s performance under real-world prevalence conditions.

## Conclusion

The present study established a reliable, non-invasive, and economically efficient nomogram based on six routine blood biomarkers to identify advanced PD. Future prospective studies incorporating genetic and imaging data will further enhance the precision of this predictive model.

## Data Availability

The raw data supporting the conclusions of this article will be made available by the authors, without undue reservation.
