# Supplementary material for: Development and external validation of a machine learning-based model for identifying advanced Parkinson’s disease
Source: Front Aging Neurosci. 2026 Jun 3;18:1867251. doi: 10.3389/fnagi.2026.1867251 (PMC13272146; doi:10.3389/fnagi.2026.1867251)
Supplement: Supplementary file 1 [file Table_1.docx]

**Supplement file**

**Table S1 Characteristics of external cohort**

| Variables | Advanced stage (N=40) | Early stage (N=40) | P value |
| --- | --- | --- | --- |
| Gender |  |  |  |
| Male | 16 (40%) | 20 (50%) | 0.500 |
| Female | 24 (60%) | 20 (50%) |  |
| Age | 68.5 ± 9.1 | 65.2 ± 9.1 | 0.115 |
| RBC | 4.6 ± 0.7 | 5.4 ± 0.8 | 0.169 |
| WBC | 7.6 ± 3.3 | 6.5 ± 1.4 | 0.059 |
| PLT | 245.2 ± 65.9 | 238.4 ± 87.1 | 0.696 |
| TB | 11.5 ± 3.1 | 9.4 ± 2.2 | <0.001 |
| IBIL | 5.5 ± 2.1 | 4.2 ± 1.5 | 0.004 |
| DBIL | 7.9 ± 3.1 | 7.0 ± 3.2 | 0.196 |
| LDH | 165.9 ± 22.3 | 199.3 ± 64.4 | 0.003 |
| ChE | 8683.4 ± 1312.2 | 7859.7 ± 1373.0 | 0.008 |
| CK | 132.2 ± 214.9 | 101.8 ± 54.6 | 0.392 |
| ALB | 42.1 ± 3.4 | 40.3 ± 3.8 | 0.032 |
